# Association of calf circumference with insulin resistance and non-alcohol fatty liver disease: the REACTION study

**DOI:** 10.1186/s12902-017-0176-4

**Published:** 2017-05-30

**Authors:** Weiwei Zhang, Zhen Yang, Yixin Niu, Xiaoyong Li, Lingfei Zhu, Shuai Lu, Hongmei Zhang, Jiangao Fan, Guang Ning, Li Qin, Qing Su

**Affiliations:** 1 Department of Endocrinology, Xinhua Hospital, Shanghai Jiaotong University School of Medicine, 1665 Kongjiang Road, Shanghai, 200092 China; 2 Department of Endocrinology, Xinhua Hospital Chongming Branch, Shanghai Jiaotong University School of Medicine, Shanghai, China; 3 Department of Gastroenterology, Shanghai Key Laboratory of Children’s Digestion and Nutrition, Xinhua Hospital, Shanghai Jiaotong University School of Medicine, Shanghai, China; 4 Department of Endocrinology and Metabolism, Key Laboratory for Endocrine and Metabolic Diseases of Ministry of Health, Ruijin Hospital, Shanghai Jiaotong University School of Medicine, E-Institute of Shanghai Universities, Shanghai, China

**Keywords:** Calf circumference, Nonalcoholic fatty liver disease, Insulin resistance, Chinese

## Abstract

**Background:**

The feature of nonalcoholic fatty liver disease (NAFLD) is pathological excessive liver lipid accumulation of subjects who without history of alcohol abuse. Calf circumference is a proxy for lower-body fat and screening method for the identification of subjects with acatastatic lipid accumulation. The objective of this study was to examine the association between calf circumference and NAFLD.

**Methods:**

The study was a cross-sectional analysis including 8850 middle-aged and elderly individuals. NAFLD was examined by hepatic ultrasound and without alcohol abuse and other liver diseases. Calf circumference was measured on the lower right leg at the point of maximal circumference.

**Results:**

The mean of calf circumference were 35.7 cm for male and 34.6 cm for female (*P* < 0.001), respectively. Compared with the lowest calf circumference quartile, the odds ratio for NAFLD in the highest quartile was 2.73 (95% CI 2.34-3.19, *P*_trend_ <0.001) after adjusted for potential cofounders. There were also significant positive correlation between calf circumference and HOMA-IR, liver enzyme levels and triglycerides. In addition, we found significant positive correlation of calf circumference with the HOMA-IR and fasting insulin level in overweight and obese subjects (BMI ≥ 24 kg/m2) but not in lean subjects (test for interaction: P both less than 0.001 for insulin and HOMA-IR).

**Conclusion:**

High calf circumference is significantly associated with elevated prevalence of NAFLD and increasing insulin resistance.

## Background

Nonalcoholic fatty liver disease (NAFLD) is characterized as pathological excessive liver lipid accumulation combined with chronic inflammatory state of subjects who without history of alcohol abuse [1]. However, the underlying mechanism of NAFLD is unclear. NAFLD is strongly linked to insulin resistance, and obesity, being prevalent in up to 95% of obese patients [2]. Certain potential mechanisms may interpret why obesity is a doughty hazard factor for the development of NAFLD [3], which include increased production of adipokines/cytokines, FFA; as well as increased levels of proinflammatory factors that contributes to abnormal hepatic metabolism. The transfer of superfluous free fatty acids (FFA) to the hepatic tissue from whole body adipose tissue lipolysis will lead to hepatic lipid accumulation. Furthermore, increased FFA levels also provoke VLDL-triglyceride production confront hyperinsulinemia [4], further increases risk of NAFLD.

Calf circumference is a substitute for lower-body adipose and credible screening measure for the identification of subjects with aberrant adipose distribution. Most recently, irresistible evidence suggests that upper body subcutaneous adipose, usually correlated with increased visceral adipose, is correlated with a metabolic disorder in different body mass indexes [5–7]. Previous studies systemic FFA concentration are primarily determined by upper-body subcutaneous fat [8, 9], however, recent studies have indicated that lower-body subcutaneous fat also play a pivotal role in accumulation of FFA in adipose tissue [10–12], indicating that leg adipose depot may play a pivotal role in risk factor pathogenesis. Increased FFA levels have been correlated with inflammation, insulin resistance, and NAFLD [13]. Several studies have established that calf circumference may be a risk factor for metabolic disorders independent of waist circumference and BMI [14, 15]. However, currently, the study on the association of calf circumference with NAFLD, and other metabolic phenotype, among Chinese individuals is scarce.

Thus, the goal of this study was to assess whether calf circumference, a infrequently used anthropometric parameter representing lean mass and peripheral fat, is correlated with NAFLD as well as other metabolic phenotypes among Chinese individuals.

## Methods

### Subjects

In 2011, China launched a national screen of Risk Evaluation of cAncers in Chinese diabeTic Individuals: a lONgitudinal (REACTION) study, which was performed among 259,657 adults, aged 40 years and older in 25 communities across mainland China, from 2011 to 2012 [16]. The material presented in this study are based on the screen from Shanghai, China [17, 18]. Subjects who had complete material about sex; age; alcohol consumption habits and smoking; BMI, and a hepatic ultrasonic examination; and a medical history including the use of medications. Participants according the followed criteria without included: 1) subjects with a medical history of known hepatic diseases such as malignancy, hepatitis, or cirrhosis; 2) subject with alcohol consumption greater than 70 g/wk for women. and140 g/wk for men. Thus, a total of 8850 participants were eventually included in this analysis. Institutional Review Board of the Xinhua Hospital approved the study protocol. All participant wrote informed consent.

### Data collection

A questionnaire was applied by health workers to collect materials such as sex, age, and medications. The history of chronic diseases and current use of medications were recorded. The smoking habit was defined as never or current (smoking regularly in the past 6 months). The information of alcohol consumption were collected. Based on the International Physical Activity Questionnaire scoring protocol, physical activity level was defined as low, moderate, or high [19].

All subjects who attend a blood sample collecton after fasting 10 h and underwent a 75-g oral glucose tolerance test (OGTT). Anthropometric information was collected by standardized protocol. Calf circumference was collected on the lower right leg at the point of maximal circumference. According to the criteria for Chinese individuals, all subjects were defined as normal weight (<24.0 kg/m^2^) or overweight or obesity (≥24.0 kg/m^2^), [20].

### Laboratory methods

Glucose oxidase method (ADVIA-1650 Chemistry System, Bayer, Leverkusen, Germany) was taken for measure plasma glucose level, high-performance liquid chromatography (BIO-RAD, D10, CA) was taken for hemoglobin A1c measurement. Fasting insulin was determined by RIA (Linco Research, St. Charles, MO). Serum C-reactive protein was determined by ELISA with Duoset kit (R&D Systems, Minneapolis, MN). Serum aspartate aminotransferase, alanine aminotransferase, and γ-glutamyltranspeptidase, total cholesterol, triglycerides, low-density lipoprotein cholesterol, high-density lipoprotein cholesterol, and creatinine were measured with an autoanalyzer (Hitachi 7080; Tokyo, Japan). According to the equation described by Matthews et al, the homeostasis model assessment of insulin resistance (HOMA-IR) was calculated [21]. Metabolic syndrome was defined using the the international consensus in the Joint Scientific Statement National Cholesterol Education Program III [22].

### Definition of NAFLD

Two ultrasonographists who were blinded to the laboratory and clinical information performed liver ultrasonic examination with a 3.5-MHz probe (Esaote Biomedica SpA, Italy). Fatty liver diagnosis based on the Chinese National Workshop on Fatty Liver and Alcoholic Liver Disease for the Chinese Liver Disease Association(2010) [23]. NAFLD was diagnosed by liver ultrasound and without other liver diseases and alcohol abuse [24].

### Statistical methods

The difference of calf circumference between subjects without and with NAFLD was analysed by the general linear model with adjusts for sex and age. The odds ratios (ORs) and 95% CIs of with NAFLD for each quartiles of calf circumference compared with the highest quartile were tested by logistic regression model, with control for sex, age, smoking, physical activity, self-reported CHD, stroke, hypertension, family history of diabetes, waist circumference, inflammatory marker, BMI and lipid profiles. The association of calf circumference with ALT, AST, GGT, A1C, fasting plasma glucose, 2-h plasma glucose, fasting insulin, HOMA-IR, triglycerides, total cholesterol, HDL-c, LDL-c, diastolic blood pressure and systolic blood pressure were evaluated by multiple linear regression model. The SPSS Statistical Package (version 15.0; SPSS Inc., Chicago, IL) were took for all statistical analyses. *P* < 0.05 was defined as statistically significant.

## Results

The mean of calf circumference were 35.7 cm for male subjects and 34.6 cm for female subjects (*P* < 0.001), respectively. Across calf circumference quartiles, subjects in higher calf circumference quartiles were more likely to be more male, more smokers, to have hypertension, to have self-reported CHD, to have self-reported stroke and to have metabolic syndrome. They also more like to have elevated liver enzyme and abnormal cardiometabolic profile (Table 1).

**Table 1:** Characteristics of study participants according to calf circumference quartiles

Quartiles of calf circumference					
	Q1: ≤32.9 cm	Q2: 33-35 cm	Q3: 35.1-36.9 cm	Q4: ≥37 cm	*P* value
*n*	2212	2213	2212	2213	
Male	513 (23.2)	620 (28.0)	748 (33.8)	954 (43.1)	<0.0001
Age (years)	56.5 ± 7.9	55.9 ± 7.9	56.1 ± 7.9	55.7 ± 8.0	0.003
Smoking, yes	316 (14.3)	361 (16.3)	389 (17.6)	458 (20.7)	<0.0001
Physical activity					0.45
Low	1588 (71.8)	1611 (72.8)	1568 (70.9)	1587 (71.7)	
Moderate	453 (20.5)	427 (19.3)	471 (21.3)	436 (19.7)	
High	171 (7.7)	175 (7.9)	173 (7.8)	90 (8.6)	
Hypertension^a^	918 (41.5)	991 (44.8)	1029 (46.5)	1199 (54.2)	<0.0001
Self-reported CHD^b^	137 (6.2)	181 (8.2)	310 (14.0)	361 (16.3)	<0.0001
Self-reported stroke^c^	62 (2.8)	82 (3.7)	102 (4.6)	175 (7.9)	<0.0001
Metabolic syndrome	911 (41.2)	1087 (49.1)	1259 (56.9)	1542 (69.7)	<0.0001
BMI (kg/m2)	22.8 ± 3.2	23.9 ± 2.9	24.9 ± 2.9	26.9 ± 7.6	<0.0001
Waist circumference (cm)	79.1 ± 11.8	82.0 ± 13.7	85.0 ± 10.8	89.8 ± 11.5	<0.0001
Fasting glucose (mmol/l)	6.25 ± 1.84	6.25 ± 1.70	6.28 ± 1.57	6.35 ± 1.67	1.12
2-h glucose (mmol/l)	8.57 ± 4.17	8.55 ± 3.83	8.71 ± 3.81	9.03 ± 3.82	<0.0001
A1C (%)	5.9 ± 1.1	5.9 ± 1.0	6.0 ± 0.9	6.1 ± 1.0	<0.0001
Fasting insulin (μU/ml)^d^	5.5 (4.0-7.6)	6.4 (4.6-8.6)	6.8 (5.0-9.4)	7.9 (5.7-10.9)	<0.0001
HOMA-IR^e^	1.51 (1.13-2.22)	1.72 (1.26-2.48)	1.85 (1.34-2.71)	2.26 (1.55-3.20)	<0.0001
ALT (U/L)	15.1 ± 11.0	16.2 ± 12.2	17.5 ± 14.0	19.6 ± 14.9	<0.0001
AST (U/L)	20.1 ± 9.3	20.4 ± 9.7	20.9 ± 12.4	21.3 ± 10.6	<0.0001
γ-GT (U/L)	25.4 ± 39.1	28.1 ± 34.7	30.2 ± 44.2	33.6 ± 35.8	<0.0001
Triglycerides (mmol/l)	1.21 (0.88-1.80)	1.31 (0.94-1.94)	1.38 (0.97-2.05)	1.52 (1.08-2.27)	<0.0001
HDL cholesterol (mmol/l)	1.31 ± 0.34	1.24 ± 0.31	1.21 ± 0.31	1.16 ± 0.28	<0.0001
CRP (μg/ml)	5.13 ± 6.74	4.82 ± 6.69	4.75 ± 5.59	5.31 ± 6.42	0.53
IL-6 (pg/ml)	1.02 (0.63–1.72)	1.04 (0.70-1.69)	1.01 (0.61-1.49)	1.08 (0.69-1.75)	0.14

Data are means ± SD, n (%), or median (interquartile range). ^a^Blood pressure ≥140/90 mmHg, or current use of anti-hypertensive medications. ^b^65 participants were excluded with a missing value for self-reported CHD. ^c^32 participants were excluded with a missing value for self-reported stroke. ^d^17 participants were excluded with a missing value of fasting insulin. ^e^21 participants were excluded with a missing value of HOMA-IR

Participants with NAFLD have higher calf circumferences than in those without NAFLD (36.1 vs. 34.2 cm, *P* < 0.0001). The NAFLD risk elevated progressively from the highest to the lowest quartiles of calf circumference with the odds ratio of 1.84 (95% CI 1.64–2.06), 3.01 (95% CI 2.68–3.38), and 5.59 (95% CI 4.94 –6.34), respectively (*P*_trend_ <0.0001) (Table 2) after control for sex and age (model 1). Further adjusts for family history of diabetesm, stroke and self-reported CHD, and behavioral factors (model 2) did not fairly change the associations. Interestingly, further control for waist circumference, inflammatory marker (model 3) only slightly reduced the magnitude of the ORs for NAFLD. Besides, the odds ratio for NAFLD was not materially weakened by additional control for lipid profiles and BMI (model 4). Although the interaction for gender was not statistically significant (*P* = 0.1542), the association in female subjects is stronger than in male subjects.

**Table 2:** Adjusted ORs (95% CI) of non-alcohol fatty liver disease according to quartiles of calf circumference

Quartiles of calf circumference					
	Q1: ≤32.9 cm	Q2: 33-35 cm	Q3: 35.1-36.9 cm	Q4: ≥37 cm	
Model 1	1 (reference)	1.84 (1.64-2.06)	3.01 (2.68-3.38)	5.59 (4.94-6.34)	<0.0001
Model 2	1 (reference)	1.70 (1.50-1.93)	2.84 (2.50-3.23)	5.07 (4.43-5.81)	<0.0001
Model 3	1 (reference)	1.30 (1.14-1.48)	1.83 (1.59-2.10)	2.85 (2.46-3.30)	<0.0001
Model 4	1 (reference)	1.29 (1.12-1.48)	1.85 (1.60-2.14)	2.73 (2.34-3.19)	<0.0001

Model 1: adjusted for age, sex (not for sex-stratified analysis). Model 2: further adjusted for physical activity (low, moderate, or high), smoking (yes/no), family history of diabetes (yes/no), self-reported CHD (yes/no), stroke (yes/no), and hypertension (yes/no) was included in the model. Model 3: further adjusted for waist circumference and inflammatory factors (CRP and IL-6, continuous variables). Model 4: further adjusted for BMI and lipid profiles (total cholesterol, triglycerides, HDL- cholesterol and LDL- cholesterol)

Calf circumference was positively correlated with ALT, AST, GGT, diastolic blood pressure, systolic blood pressure, and total cholesterol, triglycerides, and inversely correlated with HDL-cholesterol in both simple and multiple adjusted linear regression analyses (Table 3). Further control for waist circumference and BMI did not substantially alter the associations.

**Table 3:** Adjusted regression coefficients of calf circumference with metabolic parameters

	Model 1		Model 2		Model 3		Model 4	
	B (SEM)	*P*	B (SEM)	*P*	B (SEM)	*P*	B (SEM)	*P*
All (*n* = 8850)								
ALT	0.03 (0.003)	<0.0001	0.03 (0.003)	<0.0001	0.03 (0.003)	<0.0001	0.02 (0.003)	<0.0001
AST	0.01 (0.003)	<0.0001	0.01 (0.003)	<0.0001	0.01 (0.003)	<0.0001	0.01 (0.003)	0.003
GGT	0.001 (0.002)	<0.0001	0.001 (0.002)	<0.0001	0.001 (0.002)	0.001	0.001 (0.002)	0. 017
Fasting glucose (mmol/l)	0.02 (0.02)	0.252	0.01 (0.02)	0.671	-0.03 (0.02)	0.139	-0.03 (0.02)	0.059
2-h glucose (mmol/l)	0.04 (0.009)	<0.0001	0.03 (0.009)	<0.008	0.02 (0.009)	0.011	0.01 (0.009)	0.536
A1C (%)	0.15 (0.034)	<0.0001	0.11 (0.036)	0.001	0.11 (0.036)	0.002	0.05 (0.035)	0.122
^a^Fasting insulin (μU/ml)	0.06 (0.004)	<0.0001	0.06 (0.004)	<0.0001	0.04 (0.005)	<0.0001	0.03 (0.005)	<0.0001
^b^HOMA-IR	0.16 (0.013)	<0.0001	0.17 (0.015)	<0.0001	0.11 (0.015)	<0.0001	0.08 (0.014)	<0.0001
Total cholesterol (mmol/l)	0.03 (0.033)	0.445	0.01 (0.036)	0.850	0.06 (0.035)	0.092	0.08 (0.033)	0.017
LDL-c (mmol/l)	0.05 (0.044)	0.217	0.09 (0.048)	0.066	0.04 (0.046)	0.608	0.01 (0.044)	0.937
HDL-C (mmol/l)	-1.54 (0.11)	<0.0001	-1.45 (0.11)	<0.0001	-0.99 (0.11)	<0.0001	-0.78 (0.11)	<0.0001
Triglycerides (mmol/l)	0.25 (0.025)	<0.0001	0.24 (0.027)	<0.0001	0.17 (0.026)	<0.0001	0.13 (0.026)	<0.0001
Systolic blood pressure (mmHg)	0.01 (0.002)	<0.0001	0.01 (0.002)	<0.0001	0.02 (0.002)	<0.0001	0.01 (0.002)	0.0001
Diastolic blood pressure (mmHg)	0.02 (0.003)	<0.0001	0.01 (0.004)	0.083	0.01 (0.004)	0.028	0.01 (0.004)	0.005
Obesity status								
BMI < 24 kg/m^2^ (*n* = 4033)								
Fasting insulin (μU/ml)	0.03 (0.005)	<0.0001	0.03 (0.006)	<0.0001	0.01 (0.006)	0.401	-	-
HOMA-IR	0.07 (0.016)	<0.0001	0.08 (0.02)	<0.0001	0.01 (0.019)	0.479	-	-
BMI ≥ 24 kg/m^2^ (*n* = 4817)								
Fasting insulin (μU/ml)	0.08 (0.007)	<0.0001	0.07 (0.007)	<0.0001	0.05 (0.007)	<0.0001	-	-
HOMA-IR	0.22 (0.019)	<0.0001	0.19 (0.019)	<0.0001	0.14 (0.019)	<0.0001	-	-

Fasting insulin, HOMA-IR, and triglycerides were log-transformed before analysis. Model 1: adjusted for age, sex (not for sex-stratified analysis). Model 2: further adjusted for physical activity (low, moderate, or high), smoking (yes/no), family history of diabetes (yes/no), self-reported CHD (yes/no), stroke (yes/no), and hypertension (yes/no) was included in the model. Model 3: further adjusted for waist circumference and inflammatory factors (CRP and IL-6, continuous variables). Model 4: further adjusted for BMI (not for obesity status-stratified analysis). ^a^17 participants were excluded with a missing value of fasting insulin, P for interaction between calf circumference and obesity status <0.001. ^b^21 participants were excluded with a missing value of HOMA-IR; P for interaction between calf circumference and obesity status < 0.001

There were positive associations of calf circumference with HOMA-IR and fasting insulin levels in the all study population (both *P* < 0.0001 for HOMA-IR and fasting insulin levels), after adjustment for potential risk factor (Table 3). Furthermore, we found a modifying impact of obesity status on these correlations (test for interaction: both *P* < 0.0001 for HOMA-IR and fasting insulin levels). In addition, calf circumference was positively correlated with HOMA-IR and fasting insulin levels only in subjects with BMI ≥24 kg/m^2^ (both *P* < 0.0001 for HOMA-IR and fasting insulin levels) but not in subjects with BMI < 24 kg/m^2^ (*P* = 0.479 and 0.401 for HOMA-IR and fasting insulin levels, respectively) while further for stratified analyses. When subjects with BMI ≥24 kg/m^2^ were divided into calf circumference quartiles, the multivariate-adjusted geometric mean of HOMA-IR was 0.83 higher (*P* < 0.0001) and fasting insulin was 2.65 μU/ml higher (*P* < 0.0001) for the subjects in the highest quartile compared with subjects in the lowest.

## Discussion

We observed a significant correlated between calf circumference and the increased risk of having NAFLD in a large-scale population study. In addition, high calf circumference was also significantly correlated with increased insulin resistance, especially among subjects who BMI ≥24 kg/m^2^.

Recently, more studied focused extensively on body adipose distribution and metabolic abnormality. Previous studies that upper-body fat, particularly visceral adipose, is correlated with adverse metabolic phenotypes such as dyslipidemia, diabetes, insulin resistance, hypertension, and NAFLD [9, 25–27]. However, data on examination of correlation between lower-body adipose, particularly calf circumference, and metabolic phenotype, are limited, and scarce about the effect of sex and obesity status on this relationship. Currently, we demonstrate that calf circumference, as a proxy of lower-body subcutaneous adipose, is a new, detached, and pathogenic adpose depot independent of visceral fat accumulation as evaluated by the waist circumference. Although we observed that controlling for waist circumference and BMI weakens the correlation between calf circumference and NAFLD, it is worth emphasizing that correlation remained statistically significant.

Calf circumference in our study was shown to be positively associated with ALT, AST, GGT, fasting insulin, HOMA-IR, systolic blood pressure and diastolic blood pressure, total cholesterol and triglycerides, and inversely correlated with HDL cholesterol. But, in not line with the previous reports [14, 28], we have observed that calf circumference was positively correlatted with HOMA-IR and fasting insulin levels only in overweight and obese subjects but not in normal weight participants. These obesity status specific associations may partly due to the different of the amount of subcutaneous fat mass. It has well-documented that the liver is the primary site of insulin clearance in humans. Recently study found that hepatic fat accumulation is correlated with higher intrahepatic insulin exposure [29].

Increased levels of plasma FFAs and obesity status are correlated with increased VLDL triglyceride production and insulin resistance [30]. Elevated plasma FFAs levels have also been correlated with chronic systemic inflammation and oxidative stress [30, 31]. Moreover, previous study indicated visceral adipose can be considered as a marker for surfeit FFAs [32]. Previous studies have established that subcutaneous adipose is answer for a substantial proportion of systemic FFA release than visceral adipose, particularly in obese subjects [8]. Obese subjects have a larger fragment of fatty acids accumulated in subcutaneous adipose compared with lean subjects [33]. Furthermore, recent study has established that intracellular trafficking of fatty acids to triglyceride accumulation by acyl-CoA synthetase and diacylglycerol acetyltransferase may be less efficient in larger adipocytes [34]. Differences in FFA metabolism between obesity and normal weight subjects might partial explain why we only found the stronger correlation of calf circumference with insulin resistance among subjects who BMI ≥24 kg/m^2^. Given the oxidative stress and chronic low-grade systemic inflammation was the independent risk factor contribution to the development of NAFLD [35–37]. The excess free fatty acid release which caused by excessive accumulation of subcutaneous adipose might be a potential mechanism to partly explain the correlation between calf circumference and NAFLD.

As we best known, this is the first study to assess the associatin between calf circumference and NAFLD and insulin resistance in Chinese subjects. This study major strength is the analysis based on a large sample. Many potential covariates were took into account in the analysis, in order to minimize the effect of confound risk factors. Nevertheless, several limitations should be acknowledged. One of the limitation of our study is that the NAFLD diagnosis was based on ultrasound imaging, given the ultrasound examination only found subjects who at least moderate stage of the disease. Thus, we couldn’t to evaluate the association between calf circumference and mild-stage NAFLD in our study. Additionally, due to the cross-sectional nature of the present study, admittedly we cannot infer causality from our results. Limitations of epidemiological screening conditions; we could not quantify this adipose accumulation by more accurate radiographic measures.

In brief, our study indicated that increased calf circumference is correlated with an elevated risk of having NAFLD even after adjustment for potential covariates. The association of calf circumference with insulin resistance is stronger in subjects who BMI ≥24 kg/m^2^ than in lean subjects. These findings provide a relevant novel insight in the association between obesity, leg subcutaneous fat, and NAFLD. Further investigation of the underlying pathophysiological mechanism is needed to explain the positive association of leg fat with NAFLD.

## Conclusions

We have found that high calf circumference is markedly correlated with an elevated risk of having NAFLD and insulin resistance.

## Abbreviations

CVD: Cardiovascular disease
FFA: Free fatty acids
HOMA-IR: Homeostasis model assessment of insulin resistance
NAFLD: Nonalcoholic fatty liver disease
OGTT: Oral glucose tolerance test
Ors: Odds ratios
